# Impact of pharmacist-interpreted pt-qPCR technology on antimicrobial use and clinical outcomes in patients with lower respiratory tract infections: a retrospective cohort study

**DOI:** 10.3389/fphar.2025.1656557

**Published:** 2025-09-08

**Authors:** Ziyi Wang, Jing Wang, Yan Liu, Yuanyuan Wei, Yan Zhao, Shizhao Yuan, Shuai Liu, Wanyi Yin, Jing Yu

**Affiliations:** ^1^ Department of Clinical Pharmacy, The First Hospital of Hebei Medical University, Shijiazhuang, China; ^2^ The Technology Innovation Center for Artificial Intelligence in Clinical Pharmacy of Hebei Province, The First Hospital of Hebei Medical University, Shijiazhuang, China; ^3^ Department of Clinical Pharmacy, Hebei Medical University, Shijiazhuang, China; ^4^ Medical Affairs Department, The First Hospital of Hebei Medical University, Shijiazhuang, China

**Keywords:** pt-qPCR, antimicrobial stewardship, lower respiratory tract infections, rapid molecular diagnostics, real-world study, hospital-based study

## Abstract

**Objective:**

Lower respiratory tract infections (LRTIs) are a leading cause of morbidity and mortality worldwide and contribute to excessive empirical antibiotic use due to diagnostic delays. Rapid and accurate pathogen identification is essential for guiding targeted antimicrobial therapy and improving drug outcomes.

**Aim:**

This study aimed to evaluate the impact of pathogen-targeted quick multiplex PCR (pt-qPCR) compared to conventional microbiological testing on antimicrobial use and clinical outcomes in hospitalized patients with LRTIs.

**Methods:**

In this retrospective cohort study conducted at a tertiary hospital in China (March 2023–March 2024), patients with LRTIs were assigned to either a conventional testing group or a pt-qPCR group. Outcomes included pathogen detection rate, length of hospital stay (LOS), antimicrobial days of therapy (DOT), antimicrobial duration before and after testing, time to targeted therapy, mortality, and ICU transfer rate.

**Results:**

A total of 220 patients were enrolled (conventional: n = 112; pt-qPCR: n = 108). Baseline characteristics were comparable, except for higher chronic pulmonary disease prevalence (58.0% vs. 20.4%, *P* < 0.001) and lower IL-6 levels (133.22 vs. 171.28, *P* < 0.001) in the pt-qPCR group. Pathogen detection was significantly higher with pt-qPCR (94.4% vs. 53.6%, *P* < 0.001). Compared to conventional testing, the pt-qPCR group showed reduced LOS (16 vs. 16 days, *P* = 0.041), DOT (20 vs. 24 days, *P* = 0.013), post-test antimicrobial use (8 vs. 12 days, *P* < 0.001), and ICU transfer rate (31.5% vs. 49.2%, *P* = 0.006). Mortality did not differ significantly between groups. The pt-qPCR group had a higher rate of antimicrobial regimen adjustment (34.3% vs. 19.6%, *P* = 0.014) and fewer instances of escalation. A broader pathogen spectrum was detected using pt-qPCR, including 208 bacteria, 73 fungi, and 103 viruses, with 87 patients harboring multiple pathogens.

**Conclusion:**

Pharmacist-interpreted pt-qPCR significantly improves pathogen detection and optimizes antimicrobial therapy in LRTI patients. Its clinical use may enhance antibiotic stewardship, reduce ICU burden, and support precision medicine in respiratory infections.

## 1 Introduction

Lower respiratory tract infections (LRTIs) remain among the most prevalent and deadly infectious diseases worldwide. In 2019, LRTIs were responsible for over 2.6 million deaths, ranking as the fourth leading cause of mortality globally (GBD, 2019 Mental Disorders Collaborators, 2022). Beyond their clinical burden, LRTIs impose substantial economic costs on healthcare systems and patients (Zhang et al., 2016; Zhao et al., 2016; Yan et al., 2018). Early identification of causative pathogens and timely initiation of targeted therapy are critical to improving patient outcomes and reducing inappropriate antibiotic use (Buehler et al., 2016). However, traditional diagnostic methods (e.g., culture, antigen detection, serology) have long turnaround times and low sensitivity. Conventional techniques identify pathogens in only 38% of adult community-acquired pneumonia cases (Zaas et al., 2014; Jain et al., 2015). Even with combined methods, 34%–57% of pediatric and 13%–62% of adult pneumonia cases remain without a definitive microbial diagnosis (Jain et al., 2015; Emr et al., 2018; Saboktakin et al., 2019; Mani, 2018; Gadsby et al., 2016). These diagnostic limitations frequently result in empirical antibiotic overuse, delayed de-escalation, and increased risk of antimicrobial resistance.

The utility of rapid molecular diagnostics in optimizing antimicrobial therapy for respiratory infections is further supported by recent studies in this field, which highlight the value of multiplex PCR in improving diagnostic efficiency and guiding targeted treatment (Zhang et al., 2025; Hua et al., 2024). These studies, consistent with our approach, emphasize the integration of molecular testing with clinical decision-making to enhance antimicrobial stewardship. The emergence of molecular diagnostics, particularly multiplex polymerase chain reaction (PCR), presents a promising strategy to enhance both diagnostic efficiency and antimicrobial stewardship. We employed a novel pathogen-targeted rapid multiplex PCR platform (pt-qPCR) capable of detecting 96 pathogens and 8 resistance genes within 4 hours. This platform offers rapid, accurate, and cost-effective results with high reproducibility, which may directly influence prescribing behavior and reduce unnecessary antibiotic exposure. Prior studies have shown that multiplex PCR can shorten time to appropriate treatment in infectious diseases (Porter et al., 2018). Furthermore, pharmacist-led antimicrobial stewardship interventions have independently demonstrated improvements in antibiotic selection, reduced therapy duration, and enhanced clinical outcomes (Kim et al., 2021).

Given these complementary advances, there is a growing need to evaluate integrated models that combine rapid diagnostic technologies with pharmacist interpretation to optimize drug-related outcomes in LRTIs. This study aimed to evaluate the clinical utility of pharmacist-interpreted pt-qPCR testing in the diagnosis and management of LRTIs. By assessing its impact on diagnostic yield, antibiotic usage patterns, and therapeutic adjustments, we aim to generate evidence to support precision prescribing practices and inform antimicrobial stewardship policies.

## 2 Materials and methods

### 2.1 Study design

This retrospective cohort study was conducted at a tertiary teaching hospital in China from March 2023 to March 2024. Hospitalized patients with LRTIs were enrolled if they met these criteria: (1) increased or purulent airway secretions; (2) white blood cell count≥10 × 10^9^/L (neutrophils >70%); (3) fever; (4) moist rales on auscultation or inflammatory changes on chest CT (Wei et al., 2022). Inclusion also required receipt of either conventional microbiological testing or pt-qPCR during hospitalization. Exclusion criteria included: (1) age under 16 years; (2) incomplete clinical records; and (3) discharge within 48 h of admission. For patients who underwent multiple pathogen tests during a single hospitalization, only the first test result was included in the analysis. Based on the etiological diagnostic protocol implemented during hospitalization, patients were categorized into the following two groups: (1) Conventional detection group: Patients who underwent only conventional microbiological examinations; (2) pt-qPCR detection group: Patients who underwent pt-qPCR testing.

### 2.2 Clinical data collection

Clinical data were extracted from patients’ electronic medical records and included demographic characteristics such as age and gender, history of antimicrobial allergy, and comorbidities. Microbiological data included the results of all pathogen detection tests and antimicrobial susceptibility profiles conducted during hospitalization. Information on antimicrobial therapy, including drug names and the timing of initiation and discontinuation, was collected. Additional variables included length of hospital stay (LOS), in-hospital mortality, the initially suspected and confirmed sites of infection, and disease severity classification. Inflammatory biomarkers such as C-reactive protein (CRP), procalcitonin (PCT), and interleukin-6 (IL-6) were also recorded to assess the clinical status of each patient. These data were used to evaluate baseline comparability and infection-related outcomes.

### 2.3 Primary outcomes

The primary outcomes included the pathogen detection rate, LOS, antimicrobial days of therapy (DOT), duration of antimicrobial use before and after pathogen testing, time from test sampling to targeted therapy initiation, time from empirical therapy initiation to targeted therapy adjustment, and in-hospital mortality. DOT was defined as the total number of days a patient received any systemic antimicrobial agent; if more than one antibiotic was administered on the same day, each was counted as a separate DOT. The interval from empirical to targeted therapy was defined as the time between the initial administration of empirical antibiotics and the first administration of targeted therapy guided by microbiological testing. All time-related outcomes were measured in days. All reported medians for duration-related outcomes (including LOS, DOT, and duration of antimicrobial use before/after testing) were calculated per patient.

### 2.4 Secondary outcomes

Secondary outcomes included the number of antibiotic regimen adjustments, the rate of antimicrobial escalation and de-escalation, intensive care unit (ICU) admission rate, 30-day hospital readmission rate, and the appropriateness of initial empirical antibiotic therapy. Antimicrobial Adjustment: Refers to any modification made to the current antimicrobial therapy regimen based on etiological test results or clinical efficacy assessment. This encompasses changes in drug type, dosage adjustment, alteration of administration frequency, or modification of treatment duration. Escalation: The practice of switching the treatment regimen to a higher-tier (e.g., moving from the Access group to the Watch group or Reserve group within the WHO AWaRe classification) or broader-spectrum antimicrobial agent when resistant pathogens are detected or when clinical efficacy is suboptimal. Example: Changing from a second-generation cephalosporin to a carbapenem. De-escalation: The practice of narrowing the spectrum of antimicrobial therapy or stepping down to a lower-tier agent after establishing an etiological diagnosis and observing clinical improvement. Example: Switching from a combination of a broad-spectrum β-lactam and a fluoroquinolone to penicillin upon confirmation of streptococcal infection. These measures were used to assess the impact of pathogen detection methods on clinical decision-making and antimicrobial stewardship.

### 2.5 Statistical methods

All statistical analyses were conducted using Microsoft Excel 2019 (Microsoft Corp., Redmond, WA, United States) and SPSS version 26.0 (IBM Corp., Armonk, NY, United States). Continuous variables with a normal distribution were reported as mean ± standard deviation (SD) and compared using independent samples t-tests or paired t-tests, as appropriate. For non-normally distributed data, results were expressed as medians with interquartile ranges (IQR) and analyzed using the Mann–Whitney U test or the Wilcoxon signed-rank test. Categorical variables were presented as absolute counts and percentages, with group comparisons performed using the Chi-squared test or Fisher’s exact test, depending on data distribution and sample size. All statistical tests were two-tailed, and a p-value less than 0.05 was considered statistically significant.

### 2.6 Pharmacist interpretation protocol

The interpretation of pt-qPCR results was performed by clinical pharmacists from the Department of Clinical Pharmacy, First Hospital of Hebei Medical University. All participating pharmacists possessed no less than 5 years of experience in antimicrobial stewardship. Interpretation reports were delivered to clinicians within 24 h after the pt-qPCR results were issued. Primary interpretive recommendations were based on relevant guidelines and expert consensus. For complex or challenging infections, clinical pharmacists initiated real-time multidisciplinary consultations to discuss treatment strategies.

## 3 Results

### 3.1 Sample and patient characteristics

A total of 220 patients met the inclusion criteria, with 112 assigned to the conventional test group and 108 to the pt-qPCR group. Baseline demographic and clinical characteristics are summarized in Table 1. There were no statistically significant differences between the two groups in most baseline variables, indicating general comparability. However, notable exceptions included a higher prevalence of chronic lung disease in the conventional group compared to the pt-qPCR group (58.0% vs. 20.4%, P < 0.001) and a lower median IL-6 level in the conventional group (133.22 vs. 171.28, P < 0.001).

**TABLE 1:** Baseline clinical characteristics.

Characteristics	Conventional test group (112)	Pt-qPCR group (108)	P Value
Age, years, median (IQR)	73 (65–80)	70 (58.25–78)	0.085
Sex, male, n (%)	79 (70.5)	73 (67.6)	0.637
beta-lactam allergy, n (%)	14 (12.5)	8 (7.4)	0.208
Comorbidities, n (%)			
Cardiovascular disease	105 (93.8)	101 (93.5)	0.944
Diabetes	25 (22.3)	34 (31.5)	0.125
Malignant tumor	15 (13.4)	13 (12)	0.763
Chronic pulmonary disease	65 (58)	22 (20.4)	0
Chronic kidney disease	4 (3.3)	4 (3.7)	1
Chronic digestive system disease	3 (2.7)	8 (7.4)	0.108
Mental illness	1 (0.9)	1 (0.9)	1
Concurrent infection, n (%)			
Urinary tract infection	6 (5.4)	8 (7.4)	0.533
Intra-abdominal infection	2 (1.8)	1 (0.9)	1
Central nervous system infection	1 (0.9)	1 (0.9)	1
Others	0	2 (1.9)	0.24
Severity of disease, n (%)			
Septic shock	4 (3.6)	8 (7.4)	0.21
Respiratory failure	81 (72.3)	67 (62)	0.104
CRP median (IQR)	69.49 (12.11–117.02)	80.52 (26.92–130.36)	0.058
PCT median (IQR)	1.11 (0.09–0.62)	2.30 (0.10–0.90)	0.787
IL-6 median (IQR)	133.22 (0.12–4.66)	171.28 (6.78–72.55)	0
Pulmonary imaging findings, n (%)			0.832
Bilateral involvement	87 (77)	83 (76.9)	
Unilateral involvement	15 (13.4)	17 (15.7)	
Unknown	10 (8.9)	8 (7.4)	
Application of antibiotics before test, n (%)	109 (97.3)	100 (92.6)	0.108

### 3.2 Primary outcomes

Of the 220 patients included, 162 had positive pathogen detection results, 60 in the conventional test group and 102 in the pt-qPCR group. The pathogen detection rate was significantly higher in the pt-qPCR group compared to the conventional group (94.4% vs. 53.6%, P < 0.001), indicating a marked advantage of the pt-qPCR platform in improving diagnostic yield.

In addition to diagnostic performance, several clinical outcomes favored the pt-qPCR group. LOS, ICU admission rate, DOT, and the duration of antimicrobial use after pathogen testing were all significantly reduced in the pt-qPCR group (Table 2). Specifically, the average DOT was 20 days in the pt-qPCR group, 4 days fewer than in the conventional group (24 days, P = 0.013). The duration of antimicrobial therapy following pathogen testing was also significantly shorter in the pt-qPCR group (8 days vs. 12 days, P < 0.001). However, no significant differences were observed between the two groups regarding in-hospital mortality or the interval from empirical to targeted antibiotic therapy.

**TABLE 2:** Comparison of primary outcomes between conventional test and pt-qPCR.

Outcome	Conventional test group (112)	Pt-qPCR group (108)	P Value
Pathogen Detection, n (%)	60 (53.6)	102 (94.4)	<0.001
Mortality, n (%)	4 (3.6)	10 (9.3)	0.084
LOS (days), median (IQR)	16 (13–27)	16 (10–23.75)	0.041
ICU admission rate, n (%)	60 (49.2)	34 (31.5)	0.006
DOT (days), median (IQR)	24 (17–34)	20 (9–36)	0.013
Anti-pseudomonal beta-lactams, days, median (IQR)	13 (10–18)	12 (7–17)	0.123
Length of antimicrobial use before testing (days), median (IQR)	2 (1–5)	4 (1–10)	<0.001
Length of antimicrobial use after testing (days), median (IQR)	12 (9–17)	8 (4–13.25)	<0.001
Empirical therapy to targeted therapy interval (days), median (IQR)	6 (4–17)	8 (4–14.25)	0.839

### 3.3 Secondary outcomes

The application of pt-qPCR technology significantly improved the rate of antimicrobial regimen adjustments among patients with LRTIs, increasing from 19.6% in the conventional test group to 34.3% in the pt-qPCR group (P = 0.014). The number of antimicrobial escalation events was also significantly reduced in the pt-qPCR group, with 15 escalation instances compared to 32 in the conventional test group (P = 0.003). Furthermore, the use of broader-spectrum agents in initial empirical therapy was more effectively refined with pt-qPCR guidance, with the number of spectrum modifications increasing from 10 to 28 instances (P = 0.001). These findings highlight the utility of pt-qPCR in supporting precision antimicrobial management. However, no significant differences were observed between the two groups in terms of antimicrobial de-escalation rates or 30-day hospital readmission rates (Table 3).

**TABLE 3:** Comparison of secondary outcomes between conventional test and pt-qPCR.

Outcome	Conventional test group (112)	Pt-qPCR group (108)	P Value
Adjustments of antibiotic, n (%)	22 (19.6)	37 (34.3)	0.014
Escalation, n (%)	15 (13.4)	32 (29.6)	0.003
Increase spectrum of agents, n (%)	10 (8.9)	28 (25.9)	0.001
Increase application grade of agents, n (%)	7 (6.3)	14 (13)	0.09
De-escalation, n (%)	3 (2.7)	6 (5.6)	0.326
Reduce spectrum of agents, n (%)	1 (0.9)	4 (3.7)	0.206
Reduce application grade of agents, n (%)	3 (2.7)	5 (4.6)	0.493
Readmission within 30 days, n (%)	5 (4.5)	0 (0)	0.06

### 3.4 Pathogenetic detection

The spectrum and quantity of pathogens detected differed substantially between the pt-qPCR and conventional testing methods (Table 4). Using pt-qPCR, 384 pathogens were identified, comprising 208 bacteria (including 49 *Enterococcus* spp. And 57 *Streptococcus pneumoniae*), 73 fungi (predominantly 59 *Candida* spp. And 9 *Aspergillus* spp.), and 103 viruses, with *Epstein–Barr virus* (EBV) being the most common (74 detections). In contrast, conventional testing identified only 75 pathogens in the 60 positive cases, including 60 bacteria, 11 fungi, and 4 viruses. The most frequently isolated organisms in the conventional group were *Klebsiella pneumoniae* (20 isolates) and *Pseudomonas aeruginosa* (14 isolates). Notably, co-infection was common in the pt-qPCR group, with 87 patients presenting with two or more pathogens detected simultaneously, highlighting the enhanced sensitivity and breadth of the pt-qPCR platform.

**TABLE 4:** Pathogenic microorganisms isolated from the two groups.

Pathogen,n (%)	Conventional test group (112)	Pt-qPCR group (108)
Bacteria		
*Staphylococcus Aureus*	5 (4.46)	10 (9.26)
*Wnterococcus*	2 (1.79)	49 (45.37)
*Streptococcus*	0	57 (52.78)
*Escherichia Coli*	6 (5.36)	11 (10.19)
*Klebsiella Pneumoniae*	20 (17.9)	31 (28.7)
*Pseudomonas Aeruginosa*	14 (12.5)	19 (17.59)
*Baumanii*	10 (8.92)	24 (22.22)
*Stenotrophomonas Maltophilia*	3 (2.68)	7 (6.48)
Fungus		
*Candida*	11 (9.82)	59 (54.63)
*Aspergillus*	0	9 (8.33)
*Pneumocystis*	0	5 (4.63)
Virus		
*HIV*	2 (1.79)	0
*EBV*	2 (1.79)	74 (68.52)
*Human herpes virus*	0	23 (21.30)
Influenza virus	0	6 (5.56)
Others	0	3 (2.78)

## 4 Discussion

The selection and duration of empirical antimicrobial therapy for lower respiratory tract infections (LRTIs) remain a significant clinical challenge, particularly in low- and middle-income countries where multidrug-resistant (MDR) pathogens are prevalent. Although culture-based diagnostics are the current standard to guide definitive therapy, their long turnaround time often delays targeted treatment and contributes to inappropriate empirical antibiotic use and increased mortality (Dung et al., 2024; Jia et al., 2019). These challenges demonstrate the need for rapid, cost-effective, and clinically actionable diagnostic tools. However, as molecular platforms become more sensitive and detect a wider range of organisms, the lack of appropriate clinical interpretation may risk over-treatment and antimicrobial misuse (Zhuo et al., 2022a). In this study, the application of pathogen-targeted pt-qPCR combined with pharmacist-led interpretation provided a balanced approach, improving diagnostic accuracy while promoting rational antimicrobial use. Our findings confirmed that this integrated model significantly improved pathogen detection rates and contributed to reduced hospital stays.

The pt-qPCR platform demonstrated a clear advantage over conventional methods in detecting bacteria, fungi, and viruses, as well as identifying polymicrobial infections in respiratory samples. These findings are consistent with prior research on the clinical value of molecular diagnostics. For instance, Zhuo et al. used multiplex quantitative PCR (mqPCR) in 211 LRTI patients and reported a significant increase in the bacterial detection rate, from 29.9% with traditional culture to 64.5% with mqPCR, particularly in cases involving co-infections (Zhuo et al., 2022b). Similarly, Probst et al. demonstrated the effectiveness of combining real-time multiplex PCR with the carbapenem inactivation method (CIM) as a rapid and user-friendly alternative to whole-genome sequencing for surveillance of carbapenemase-producing *Enterobacterales* (Probst et al., 2021). In pediatric populations, Wang et al. found that GeXP-based PCR and multiplex qPCR had a significantly higher detection rate compared to routine diagnostics (76.09% vs. 36.13%, *P* = 0.004), suggesting that molecular tools can meaningfully enhance the diagnosis and treatment of LRTIs in children (Wang et al., 2021).

While total antimicrobial duration was shorter in the pt-qPCR group, pre-test therapy was longer. This likely reflects clinicians’ reluctance to use higher-cost diagnostics upfront, preferring empirical therapy or cheaper conventional tests. Nonetheless, the reduction in antimicrobial duration post-testing, combined with shorter LOS and reduced ICU utilization, suggests that pt-qPCR may offer long-term economic advantages (Darie et al., 2022). Furthermore, pt-qPCR significantly increased the rate of antimicrobial regimen adjustments. This technology enabled timely shifts to targeted therapy, improving infection control while minimizing unnecessary broad-spectrum antimicrobial use. Our findings align with prior research in this journal demonstrating that rapid pathogen detection technologies, when combined with structured clinical interpretation, can significantly reduce inappropriate antimicrobial use (Zhang et al., 2023). This consistency reinforces the methodological robustness of leveraging molecular diagnostics for LRTI management. These benefits are critical in the fight against antimicrobial resistance. Notably, while escalation rates differed significantly between groups, de-escalation rates did not. This may reflect a conservative approach in initial empirical therapy, where clinicians prioritize broad coverage in severely ill patients (Claeys et al., 2020).

These findings suggest that pt-qPCR testing, when integrated with pharmacist interpretation, contributes directly to improved drug outcomes by reducing unnecessary antimicrobial exposure and promoting early targeted therapy. This has the potential to reduce adverse drug reactions and slow the development of antimicrobial resistance in hospital settings. From a health policy perspective, this study supports the integration of pharmacist-led diagnostic interpretations into hospital antimicrobial stewardship programs. The adoption of rapid molecular testing as a routine clinical tool could be incentivized in formulary and reimbursement policies to promote evidence-based antibiotic prescribing. Although the upfront cost of pt-qPCR testing may be higher, the associated reductions in total antibiotic use, LOS, and ICU admissions suggest improved cost-effectiveness, particularly in resource-constrained settings where efficient drug use is critical.

Despite its promising findings, this study has several limitations. First, it was conducted at a single center with a modest sample size and limited duration, which may affect generalizability. Second, While pt-qPCR shows clinical value with pharmacist interpretation, its scalability in resource-limited settings faces challenges: limited pharmacists may delay or suboptimally interpret complex results (e.g., polymicrobial infections); underdeveloped AMS infrastructure hinders workflow integration; and upfront costs are prohibitive. To address these challenges, several strategies may enhance feasibility. Feasibility can be enhanced via algorithm-based decision tools to reduce pharmacist reliance, telepharmacy to address staffing shortages, phased implementation prioritizing high-risk patients, and policy support (e.g., subsidies, integration into national AMS frameworks) to adapt to resource constraints.

## 5 Conclusion

Pharmacist-interpreted pt-qPCR significantly improved pathogen detection and reduced hospital stay, antimicrobial duration, and ICU admissions in patients with LRTIs. These findings support its role in promoting targeted antibiotic use and enhancing antimicrobial stewardship. pt-qPCR has strong potential for routine clinical integration and policy adoption to optimize drug outcomes and resource use.
